# Transition of healthy to diseased synovial tissue in rheumatoid arthritis is associated with gain of mesenchymal/fibrotic characteristics

**DOI:** 10.1186/ar2073

**Published:** 2006-10-31

**Authors:** Marjan MC Steenvoorden, Tanja CA Tolboom, Gabri van der Pluijm, Clemens Löwik, Cornelis PJ Visser, Jeroen DeGroot, Adriana C Gittenberger-DeGroot, Marco C DeRuiter, Bert J Wisse, Tom WJ Huizinga, René EM Toes

**Affiliations:** 1 Department of Rheumatology, Leiden University Medical Center, Albinusdreef 2, 2333 ZA Leiden, The Netherlands; 2 TNO Quality of Life, Business Unit Biomedical Research, Zernikedreef 9, 2333 CK Leiden, The Netherlands; 3 Department of Endocrinology, Leiden University Medical Center, Albinusdreef 2, 2333 ZA Leiden, The Netherlands; 4 Department of Orthopaedics, Rijnland Hospital, Simon Smitweg 1, 2353 GA Leiderdorp, The Netherlands; 5 Department of Anatomy and Embryology, Leiden University Medical Center, Albinusdreef 2, 2333 ZA Leiden, The Netherlands

## Abstract

The healthy synovial lining layer consists of a single cell layer that regulates the transport between the joint cavity and the surrounding tissue. It has been suggested that abnormalities such as somatic mutations in the p53 tumor-suppressor gene contribute to synovial hyperplasia and invasion in rheumatoid arthritis (RA). In this study, expression of epithelial markers on healthy and diseased synovial lining tissue was examined. In addition, we investigated whether a regulated process, resembling epithelial to mesenchymal transition (EMT)/fibrosis, could be responsible for the altered phenotype of the synovial lining layer in RA. Synovial tissue from healthy subjects and RA patients was obtained during arthroscopy. To detect signs of EMT, expression of E-cadherin (epithelial marker), collagen type IV (indicator of the presence of a basement membrane) and α-smooth muscle actin (α-sma; a myofibroblast marker) was investigated on frozen tissue sections using immunohistochemistry. Fibroblast-like synoviocytes (FLSs) from healthy subjects were isolated and subjected to stimulation with synovial fluid (SF) from two RA patients and to transforming growth factor (TGF)-β. To detect whether EMT/fibrotic markers were increased, expression of collagen type I, α-sma and telopeptide lysylhydroxylase (TLH) was measured by real time PCR. Expression of E-cadherin and collagen type IV was found in healthy and arthritic synovial tissue. Expression of α-sma was only found in the synovial lining layer of RA patients. Stimulation of healthy FLSs with SF resulted in an upregulation of α-sma and TLH mRNA. Collagen type I and TLH mRNA were upregulated after stimulation with TGF-β. Addition of bone morphogenetic protein (BMP)-7 to healthy FLS stimulated with SF inhibited the expression of α-sma mRNA. The finding that E-cadherin and collagen type IV are expressed in the lining layer of healthy and arthritic synovium indicates that these lining cells display an epithelial-like phenotype. In addition, the presence of α-sma in the synovial lining layer of RA patients and induction of fibrotic markers in healthy FLSs by SF from RA patients indicate that a regulated process comparable to EMT might cause the alteration in phenotype of RA FLSs. Therefore, BMP-7 may represent a promising agent to counteract the transition imposed on synoviocytes in the RA joint.

## Introduction

Although the synovial lining has been described as a mesenchymal tissue because it lacks several epithelial properties, such as tight junctions and desmosomes [1], its function and morphology resemble that of epithelial tissues. Epithelial tissues cover or line body surfaces, forming the surface of the skin, the epidermis, the linings of body cavities (mesothelium) and internal lining of the digestive system and glands. The function of epithelium is to form a barrier between the external and internal environment and to regulate transport between the cavity it encloses and the adjacent tissue by facilitating transport and secretion [2]. In the normal state, the function of the synovial tissue is to facilitate skeletal movement by the maintenance of a fluid-filled space around cartilage or tendon surfaces. The fibroblast-like synoviocytes are responsible for the excretion of factors such as hyaluronan into the synovial fluid, for clearance of intra-articular debris and regulation of immunological events [1,3].

In rheumatoid arthritis (RA) synovial hyperplasia and inflammation play a prominent role. While influx of inflammatory cells such as macrophages are important in the inflammation of the tissue [4], proliferation of fibroblast-like synoviocytes (FLSs) seems to be a major cause of the hyperplasia of the synovial tissue [5,6]. In addition, the data indicate that FLSs could play a role in cartilage degradation, as they are found at sites of cartilage degradation in RA and are able to degrade and invade cartilage when co-implanted into a SCID mouse [7].

It has been proposed that the changes in the RA synovium are a random process, caused by, for example, altered expression of p53 [8,9]. In RA, overexpression of p53 has been found, often together with somatic mutations in the gene encoding p53, some of which result in an inactive p53 protein [10-12]. For these reasons, it has been suggested that mutations in genes involved in the control of cell-cycle and survival, like that encoding p53, are involved in the deranged behaviour of FLSs in RA synovium.

The changes that occur in the synovial lining during development of RA, however, resemble the changes of the peritoneal lining during chronic ambulatory dialysis. During chronic ambulatory dialysis, the epithelial cells that form the peritoneal lining become hyperplastic and show a transformed mesenchymal phenotype (myofibroblast phenotype). These changes are induced in a process called epithelial-to-mesenchymal transition (EMT) [13].

Fibrosis, abnormal wound healing, is another process in which EMT can play a role. Myofibroblasts, expressing α-smooth muscle actin (α-sma), are formed from fibroblasts or from epithelial cells by EMT. The myofibroblasts are responsible for matrix deposition and wound contraction and, after normal wound healing, will die by apoptosis or transform into quiescent cells [14,15]. During fibrosis however, the presence of myofibroblasts persists, leading to overproduction of extracellular matrix, and eventually to loss of function of the organ [16]. This accumulation of extracellular matrix is also found in RA, indicating that both the gain of invasiveness of RA synoviocytes as well as the increased matrix production seen in fibrosis represent important pathophysiological events mediated by synoviocytes.

During EMT, the intercellular adhesion molecule E-cadherin appears to play a central role. Transfection of a mesenchymal cell line with E-cadherin resulted in the transdifferentiation into an epithelial cell, while inhibition of E-cadherin expression leads to transdifferentiation from epithelial cells to mesenchymal cells [17]. Together with alteration of cell morphology, other mesenchymal characteristics, such as an invasive motility, the expression of α-sma and vimentin, appear [18]. The invasive motility plays an important role in metastasis of tumours into surrounding tissues, a process resembling invasion of FLSs into cartilage.

Plasticity resulting from cells shifting between mesenchymal phenotypes is discernible either by EMT only or by the reverse process, called mesenchyme-to-epithelium transition. Both processes have emerged as a fundamental principle for reprogramming of gene transcription and as a major determinant of stem cell fate in development and tissue homeostasis. While transforming growth factor (TGF)-β has been identified as one of the main inducers of EMT during development and fibrotic disorders, another member of the TGF-β super family, bone morphogenetic protein (BMP)-7 (also called osteogenic protein 1), is involved in the maintenance of the epithelial phenotype by induction of mesenchyme-to-epithelium transition [19].

Although TGF-β is the most well known inducer of EMT, EMT can also be induced by S100 calgranulins and advanced glycation endproducts (AGEs) or by the proteolytic digestion of the basement membrane [20-24]. Levels of both TGF-β, AGEs and S100 calgranulins are increased in the synovial fluid of RA patients and, therefore, the synovial lining is exposed to inducers of EMT [20,25].

The aim of this study was to investigate whether the synoviocytes from RA patients may have undergone a regulated process resembling EMT/fibrosis (as was previously suggested by Zvaifler [26]). This is also of relevance as recent *in vivo *and *in vitro *studies have shown that renal fibrosis can be reversed by administration of BMP-7 [24], indicating that, if EMT does play a role in RA, BMP-7 might be an interesting therapeutic drug.

## Materials and methods

### Patients, human tissue samples and synovial fluid

Synovial tissue was obtained from healthy subjects and RA patients during arthroscopy of the knee. All RA patients met the 1987 criteria of the American College of Rheumatology. Healthy subjects were people that underwent arthroscopy because of ruptured menisci or ligaments. None of these healthy subjects had a history of inflammatory joint disease. Before healthy synovial tissue was collected, permission according to the international conference on harmonisation in Helsinki was obtained. Synovial tissue was obtained at two different hospitals; at both locations permission of authorized ethical commissions was obtained. All patients and healthy subjects gave informed consent. Synovial tissues were collected during the arthroscopy, frozen in Tissue-Tek OCT compound (Sakura Finetek, Zoeterwoude, the Netherlands) and cut into 5 μm slices using a cryotome (Leica CM 1900).

In addition, some of the healthy synovial tissue was digested with collagenase IA (1 mg/ml; Sigma, St Louis, MO, USA) directly after arthroscopy. Cells were separated from tissue debris by filtration through a 200 μm filter (NPBI, Emmer-Compascuum, the Netherlands) and cultured in 25 cm^2 ^flasks (Cellstar, Greiner, Alphen aan de Rijn, the Netherlands) with Iscove's Modified Dulbecco's medium (BioWhittaker, Versiers, Belgium) supplemented with glutamax (GibroBRL, Paisley, UK) and penicillin and streptomycin (Boehringer, Mannheim, Germany) with 10% foetal calf serum (GibcoBRL) at 37°C in the presence of 5% CO_2_. When cells had reached confluence, they were detached with 0.025% trypsin and split in a 1:3 ratio. Fourth passage cells were used for stimulation experiments and consisted of more than 95% FLSs as evaluated with light microscopy.

Synovial fluid (SF) was obtained from swollen joints from RA patients. It was collected in a 50 ml tube, cells were removed and aliquots were kept at -80°C. For experiments, SF was diluted with medium.

### Haematoxylin eosin staining

Sections from all tissues were first stained with haematoxylin and eosin to evaluate the morphology of the synovial tissue.

### Immunohistochemistry of human cryo-sections

Immunohistochemistry was performed following standard protocols. In short, samples were thawed for 30 minutes at room temperature and then fixed with acetone for 10 minutes. During acetone dehydration, endogenous peroxidase was blocked with 0.3% H_2_O_2_. Before applying the first antibody, samples were blocked with 10% normal human serum in PBS containing 1% bovine serum albumin for 1 h. After this blocking step, anti-E-cadherin (clone 4A2C7, Zymed, San Francisco, USA) was applied diluted 100× in PBS/1% bovine serum albumin and incubated overnight at room temperature. The next day sections were thoroughly washed with PBS. For E-cadherin staining, sections were incubated with a second antibody: rabbit anti-mouse (Dako, Heverlee, Belgium) for 30 minutes at room temperature followed by 30 minutes incubation with alkaline phosphatase anti-alkaline phosphatase (Dako). Staining was visualized using a 4-nitro blue tetrazolium chloride/5-bromo-4-chloro-3-indolyl-phosphatase (NBT/BCIP) reaction containing 0.33 mg/ml NBT (stock: 50 mg/ml in 70% dimethylformamide; Roche, Mannheim, Germany), 0.17 mg/ml BCIP (stock: 50 mg/ml in 100% dimethylformamide; Roche), 3.33 mM levamisole (Sigma), 100 mM Tris, 100 mM NaCl, 5 mM MgCl_2_·6H_2_O pH 9.5. These sections were incubated for 10 minutes at room temperature and the reaction was stopped in H_2_O. Sections were counterstained with 1% light green solution (Sigma). Samples were mounted with glycerol glycine and covered with a coverslip.

For α-sma staining, sections were prepared as for E-cadherin. α-Sma antibody (clone 1A4, DAKO Cytomation, Heverlee, Belgium) was biotinylated using the DAKO animal research kit (DAKO ARK, Dako) before application and incubated overnight at 4°C. The next day sections were thoroughly washed with PBS and incubated with streptavidin-horse radish peroxidase (HRP) for 15 minutes at room temperature. NovaRed (Vector Laboratories, Burlingame, CA, USA) was used for HRP detection. Sections were counterstained with Mayers haematoxylin and mounted and covered as described above.

For collagen IV staining, sections were again prepared as for E-cadherin and α-sma staining and then incubated overnight with a rabbit anti-human antibody against collagen type IV (200 μg/ml; Santa Cruz, CA, USA). Next, a biotinylated secondary antibody, goat anti-rabbit (Kirkegaard and Perry Laboratories, Gaithersburg, MD, USA) was used and subsequently conjugated with streptavidin-HRP and detected using NovaRed.

Double stainings for collagen type IV and FLS marker CD55 were performed after preparation of the sections as described above. Sections were incubated overnight with a rabbit anti human antibody against collagen type IV (200 μg/ml, Santa Cruz) and subsequently for 2 h with anti-CD55 mouse anti-human antibody (clone BRIC 110, CLB, Amsterdam, the Netherlands). Next, both a biotinylated goat anti-rabbit and an alkaline phosphatase labelled goat anti-mouse (DAKO) secondary antibody were used. The biotinylated antibody was subsequently conjugated with streptavidin-HRP and detected using NovaRed. In addition, the CD55 staining was detected by NBT/BCIP.

### Real-time PCR on cultured fibroblast-like synoviocytes

FLSs from RA patients and healthy controls were plated in a 24-well flat bottom plate at a density of 100,000 cells per well. After 72 h, cells were lysed for RNA isolation. RNA was isolated using the Qiagen RNeasy kit (Qiagen, Hilden, Germany) and subsequently converted to cDNA (AMV reverse transcriptase, Roche, Penzberg, Germany) and subjected to real-time PCR amplification. For detection of collagen type IV mRNA expression, cDNA was amplified using specific primers for collagen type IV and the housekeeping gene for β2-microglobulin (β2M) (Table 1). The total reaction volume of 25 μl contained 1× PCR buffer (Applied Biosystems, Nieuwerkerk aan den Ijssel, the Netherlands), 0.4 mM of each dNTP, 2.5 mM MgCl^2+^, 500 nM of each primer and 1 unit of AmpliTaq Gold polymerase (Applied Biosystems) together with 10 μl 5× diluted cDNA. PCR was performed using an iCycler (Bio-Rad laboratories Inc, Hercules, CA, USA) and consisted of a 4 minute interval at 94°C followed by 30 cycles of 95°C for 30 s, 56°C or 60°C for 45 s, and 72°C for 30 s for β2M and collagen type IV, respectively. An aliquot of 15 μl of each sample was subjected to electrophoresis in a 2% agarose gel containing ethidium bromide.

### Stimulation of healthy FLSs with TGF-β and SF

One day prior to stimulation, healthy FLSs were plated in a 24-well flat bottom plate at a density of 100,000 cells per well. One hour before stimulation, cells were washed with PBS and incubated in serum free medium. Cells were stimulated in medium with 0.1% lactalbumin hydrolysate (as serum replacement) alone (as a control) or in medium with 0.1% lactalbumin hydrolysate containing either 1 ng/ml TGF-β or 10× diluted synovial fluid for an additional 48 h. At this point, cells were lysed for RNA isolation. RNA was isolated using the Qiagen RNeasy kit and subsequently converted to cDNA (AMV reverse transcriptase, Roche, Penzberg, Germany) and subjected to real-time PCR amplification. cDNA was amplified using specific primers and specific beacons for collagen type I α2 chain, α-sma, telopeptide lysyl hydroxylase (TLH), and the housekeeping gene for β2M (Tables 1 and 2). The total reaction volume of 25 μl contained 1× PCR buffer (Applied Biosystems), 0.4 mM of each dNTP, 2.5 mM MgCl^2+^, 500 nM of each primer and beacon and 1 unit of AmpliTaq Gold polymerase (Applied Biosystems) together with 10 μl 5× diluted cDNA. PCR was performed in an ABI PRISM^® ^7700 sequence detection system and consisted of a 5 minute interval at 95°C followed by 40 cycles of 95°C for 30 s, 56°C for 40 s, and 72°C for 30 s. Data were analysed using Sequence Detector version 1.7 software (Applied Biosystems).

### Statistical analysis

For statistical analysis, means and standard deviations were calculated. Differences between conditions were tested for statistical significance using the Kruskall-Wallis test with a *post hoc *Mann-Whitney U-test. Differences were considered statistically significant at p < 0.05. All statistical analyses were performed using SPSS 11.5 Software (SPSS, Chicago, IL, USA).

## Results

### Expression of epithelial and mesenchymal markers on synovial tissue

**Figure 1:**
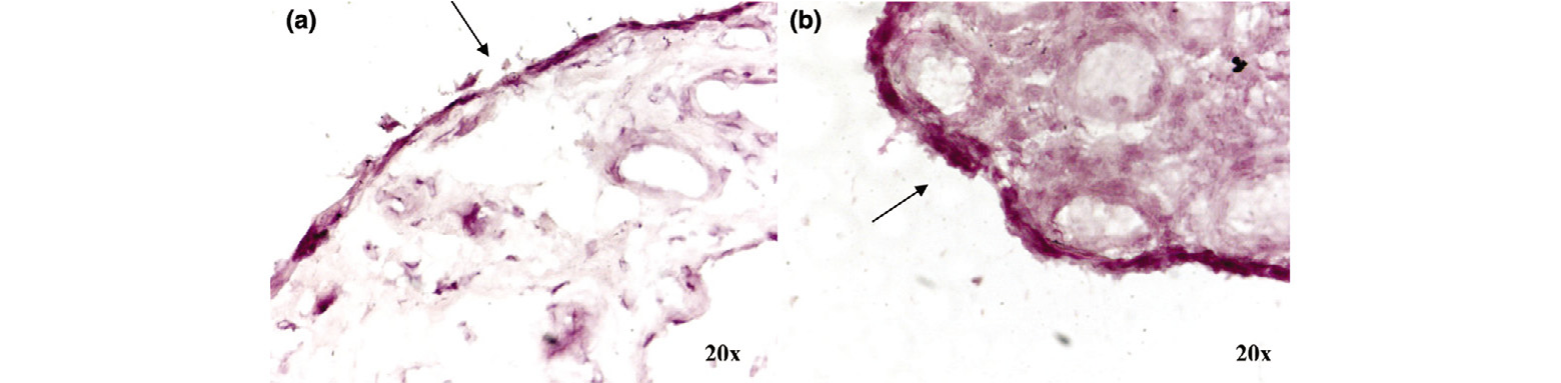
Haematoxylin and eosin staining on healthy and arthritic synovial tissue. Haematoxylin and eosin staining was performed on synovial biopsies from **(a) **healthy subjects and **(b) **Rheumatoid arthritis patients. Arrows indicate synovial lining.

**Figure 2:**
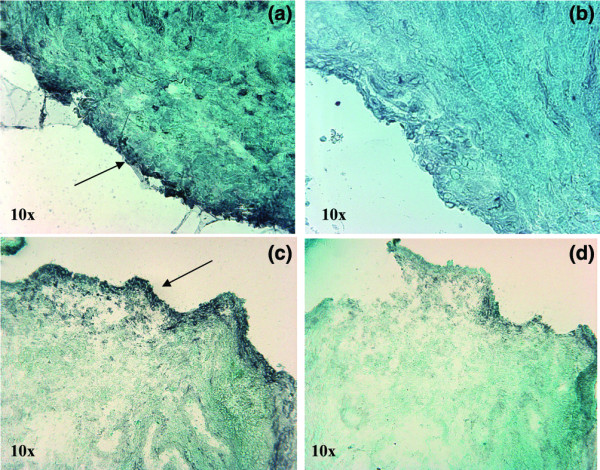
E-cadherin staining in healthy and arthritic synovial tissue. Synovial biopsies from **(a) **healthy human subjects and **(c) **rheumatoid arthritis patients show positive E-cadherin staining in the lining layer. Relative isotype controls are shown in **(b,d)**. Arrows indicate E-cadherin positive cells from the lining layer.

As previously described, staining of human synovial tissue with haematoxylin and eosin showed an increase in the number of cell layers of the synovial lining of RA patients (Figure 1). E-cadherin staining was found in the healthy synovium, especially in the lining layer (Figure 2). In arthritic synovial tissue, the E-cadherin expression pattern was more widely spread, possibly due to hyperplasia (found in all patients). The presence of E-cadherin staining indicates that most synoviocytes displayed epithelial properties.

**Figure 3:**
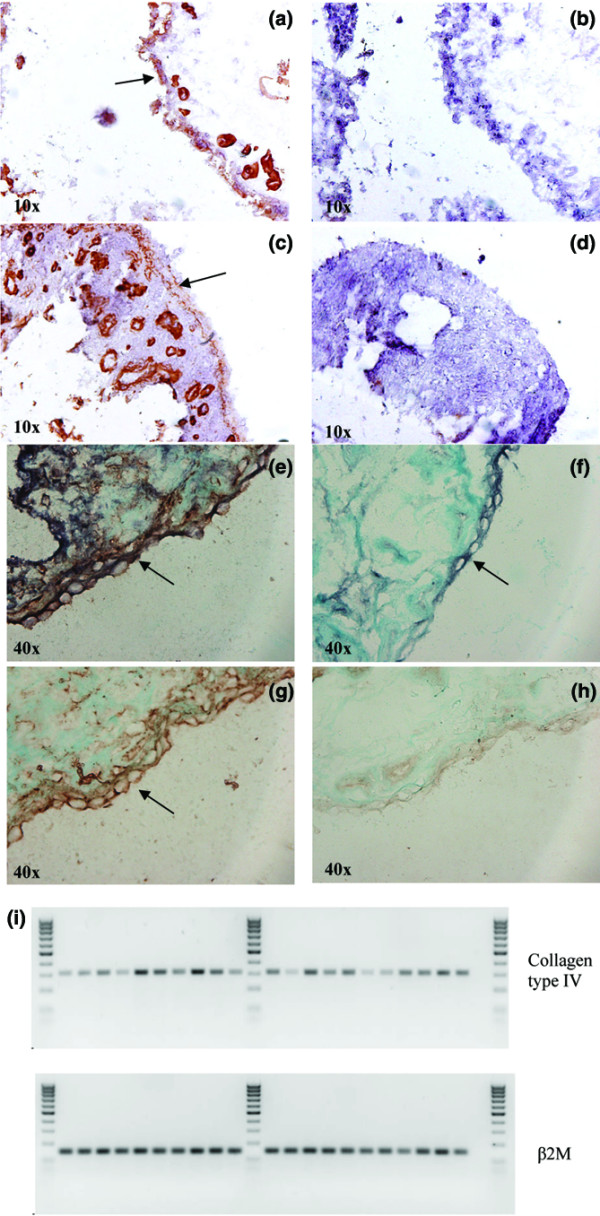
Collagen type IV staining in healthy and arthritic synovial tissue. Collagen type IV was found in the lining layer of synovial biopsies from **(a) **healthy human subjects and **(c) **rheumatoid arthritis (RA) patients. Relative isotype controls are shown in **(b,d)**. Double staining was performed to reveal the cellular origin of the type IV collagen. **(e) **Double staining was found in the lining layer. For a clearer picture, succeeding slices were stained with **(f) **CD55 or **(g) **collagen type IV. **(h) **Isotype control. Arrows indicate collagen IV, CD55 or double staining in the lining layer. **(i) **PCR results: top, collagen type IV mRNA expression in fibroblast-like synoviocytes from 10 RA patients and 11 healthy controls; bottom, β2-microglobulin (β2M) mRNA expression in the same samples.

Another epithelial property is the presence of a basal membrane, consisting of collagen IV and laminin. Collagen IV was present all along the lining layer of both healthy and arthritic synovial tissue, indicating the presence of a (partial) basement membrane (Figure 3a). Double staining with FLS marker CD55 showed co-localisation of collagen type IV and CD55 expression (Figure 3e,f). In addition, expression of collagen type IV mRNA was found in cultured FLSs from both RA patients and healthy controls (Figure 3i), indicating that FLSs are able to produce collagen type IV.

**Figure 4:**
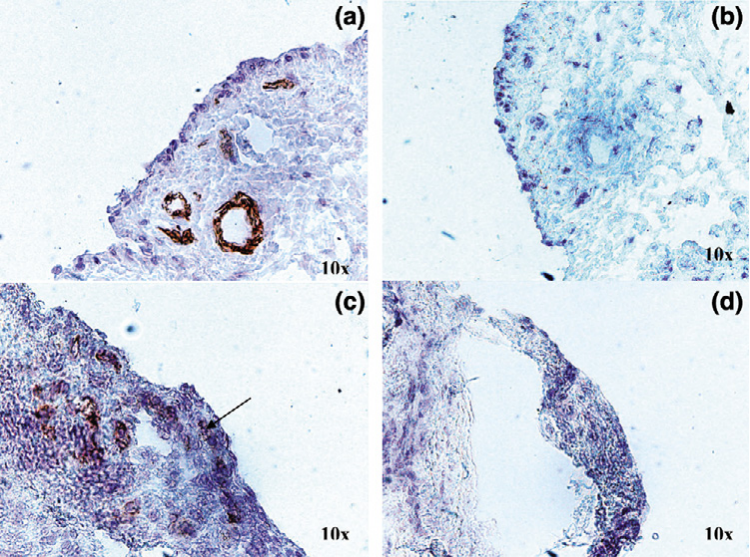
α-Smooth muscle actin (α-sma) staining in healthy and arthritic synovial tissue. **(a) **No α-sma staining was found in the lining layer of healthy synovial tissue. **(c) **In synovial biopsies from rheumatoid arthritis patients, cells positive for α-sma expression were found in the lining layer. Relative isotype controls are shown in **(b,d)**. Arrows indicate α-sma positive cells from the lining layer.

Additional markers were tested to further define the epithelial and mesenchymal properties of the synoviocytes in the lining layer. α-Sma is a myofibroblast marker often used to identify myofibroblasts after EMT. In healthy synovium, α-sma expression was only found in blood vessels. In contrast, in synovial tissue from four out of eight patients with RA, α-sma expression was also found in the lining layer, suggesting that the lining of the synovium in RA patient contains myofibroblasts (Figure 4). Together, these data indicate that several epithelial markers are present in healthy and RA synovial tissue. In addition, the presence of α-sma in human RA synovial tissue indicates that an EMT-like/fibrotic process has occurred.

### Different effects of TGF-β, SF and BMP-7 stimulation on collagen type I α2 chain, α-sma and TLH expression in FLSs

**Figure 5:**
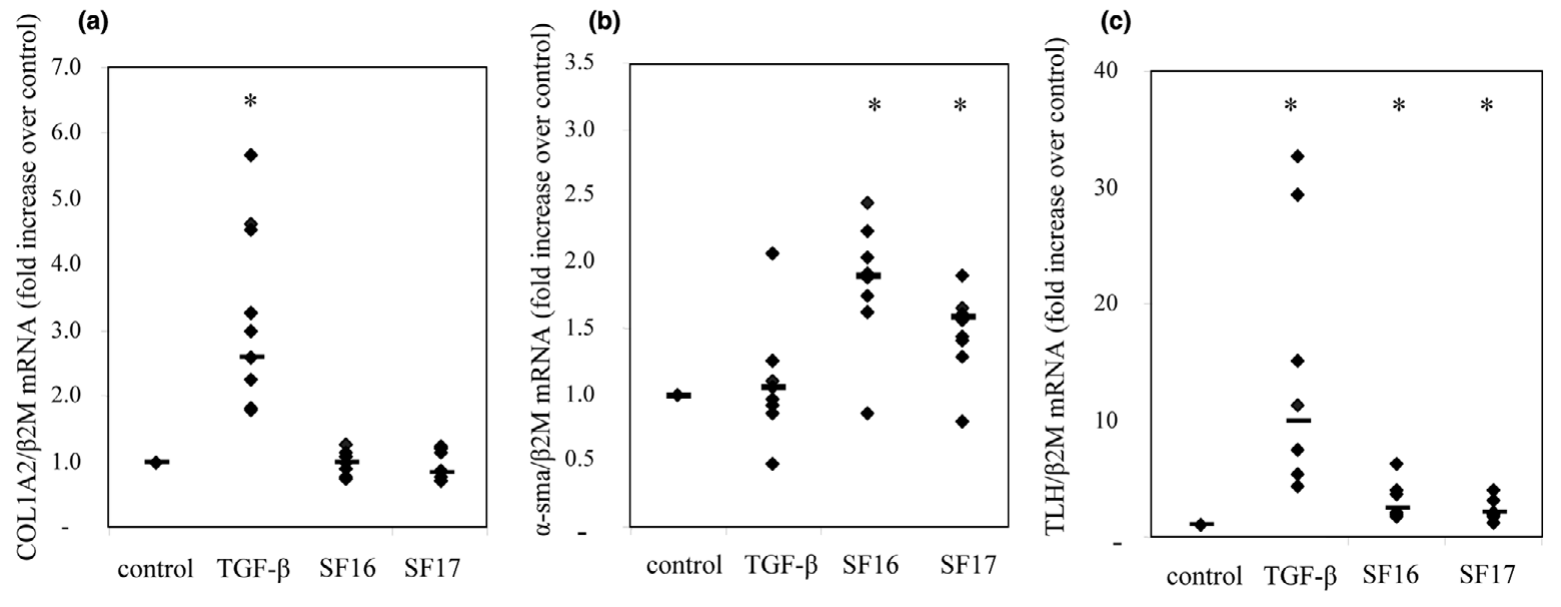
Expression of collagen type I, α-smooth muscle actin (α-sma) and telopeptide lysylhydroxylase (TLH) in healthy fibroblast like synoviocytes (FLSs) upon stimulation with transforming growth factor (TGF)-β and synovial fluid (SF). **(a) **Expression of collagen type I alpha 2 chain (COLIA2)/β2-microglobulin (β2M) mRNA, **(b) **α-sma/β2M mRNA and **(c) **TLH/β2M mRNA were measured after stimulation of healthy FLSs with 1 ng/ml TGF-β or 1/10 diluted SF from rheumatoid arthritis patients. TGF-β increased COLIA2 expression, but had no effect on α-sma expression. Stimulation of healthy FLSs with synovial fluid from RA patients (RASF) did not lead to an increase in COLIA2/β2M but did induce α-sma/β2M mRNA expression. TLH expression was induced by both TGF-β and SF. Horizontal lines indicate the median; diamonds indicate FLSs from individual subjects. * indicates P < 0.05.

To investigate whether synovial fluid is able to induce an alteration in phenotype and expression of healthy FLSs, we stimulated FLSs obtained from 10 healthy subjects with TGF-β and SF from two RA patients. Expression of three markers often upregulated in fibrotic processes and after EMT, collagen type I α2 chain, α-sma and TLH mRNA, was measured and corrected for the expression of the housekeeping gene β2M. Upon stimulation with SF from two different RA patients, expression of collagen I remained stable, while expression of α-sma was modestly increased (1.8 ± 0.5-fold with SF16 and 1.4 ± 0.3-fold with SF17 (mean ± standard deviation), *n *= 10, p < 0.001 for both SFs) (Figure 5). This indicates an alteration from healthy FLSs to a phenotype resembling that of differentiated myofibroblasts, a cell type that shows increased proliferation and is capable of migration and invasion. In addition, we found an upregulation in TLH, an enzyme responsible for the increased crosslinking of collagen in fibrotic tissue [27], after stimulation of healthy synoviocytes with SF (3.4 ± 1.7-fold induction with SF16 and 2.7 ± 1.3-fold with SF17, *n *= 10, p < 0.001 for both SFs) (Figure 5). This indicates that SF might also induce collagen crosslinking in synovial tissue, thereby reducing the susceptibility of collagen for degradation.

To study whether the effects of SF on α-sma expression could be induced by TGF-β in the SF, healthy FLSs were stimulated with 1 ng/ml TGF-β (the level of total TGF-β normally found in RA SF [28]) and expression of collagen I, α-sma and TLH was measured. Interestingly, a discrepancy between stimulation with TGF-β and SF was found. Upon stimulation with TGF-β, an increased expression of collagen I (3 ± 1.4-fold increase, *n *= 10, p < 0.001) and TLH (14.5 ± 10.8-fold increase, *n *= 8, p < 0.001) were found. No effect of TGF-β on α-sma expression was found. This could indicate that, although this concentration of TGF-β is able to induce enzymes responsible for modification of extracellular matrix in healthy FLSs, it is unable on its own to induce an alteration towards myofibroblasts after 48 hours. Using an ELISA for active TGF-β1, we found only very low levels of TGF-β1 in our synovial fluid (data not shown), indicating that, indeed, the effect of SF on healthy FLSs might not be mediated by TGF-β1 in the SF from these two donors. Although the differences found with real-time PCR are modest, they are highly reproducible: 10 donors cultured on different days and assayed by real-time PCR on different days show similar results when cultured with TGF-β and SF. In addition, culture of cells from two patients and subsequent analysis by real-time PCR was repeated after a few weeks and, again, similar results were found. Together, these data indicate that, even within this relatively short time-frame, SF and TGF-β are able to induce EMT/fibrotic markers in healthy synoviocytes.

**Figure 6:**
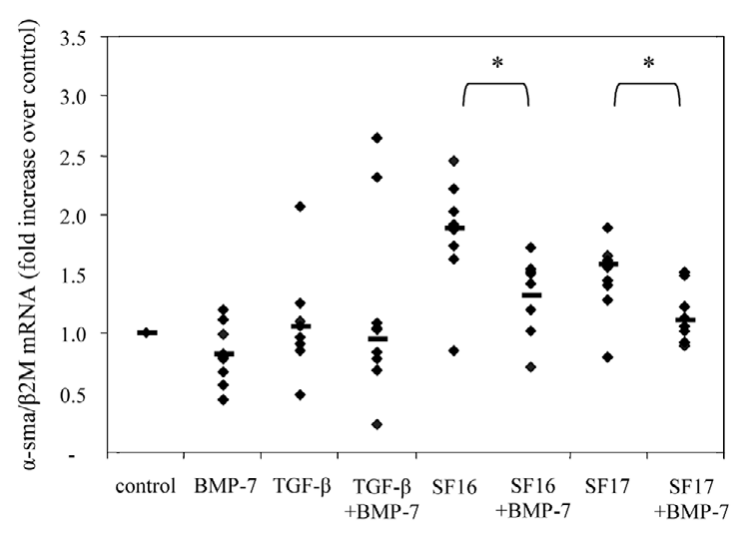
Expression of α-smooth muscle actin (α-sma) upon stimulation with transforming growth factor (TGF)-β or synovial fluid (SF) in combination with bone morphogenetic protein (BMP)-7. BMP-7 treatment of healthy FLSs inhibited the induction of α-sma mRNA by SF16 and SF17 in FLS from all donors, Horizontal lines indicate the median; diamonds indicate FLSs from individual subjects. * indicates P < 0.05.

BMP-7 has been described as an inhibitor of EMT and fibrosis and can induce expression of epithelial markers. These opposing effects of BMP-7 on TGF-β signalling are of interest as they could inhibit the fibrotic process evoked by SF and/or TGF-β. We aimed to examine whether addition of BMP-7 to synoviocytes obtained from healthy donors would inhibit the expression of the fibrotic marker α-sma (Figure 6). Administration of BMP-7 to healthy FLSs *in vitro *had no effect on α-sma expression in unstimulated cells (0.8 ± 0.2-fold expression, *n *= 10, p = 0.06) (Figure 6). Administration of BMP-7 together with SF inhibited the effects of SF, as shown by the observation that expression of α-sma was less pronounced compared to controls (inhibition of 1.8-fold compared to 1.3 for SF16 and 1.4 to 1.2 for SF17, *n *= 10, p < 0.01).

Together, these data indicate that TGF-β and synovial fluid from RA patients are able to induce upregulation of EMT/fibrotic markers on mRNA levels, which can be partially suppressed by BMP-7.

## Discussion

This study indicates that the cells of the synovial lining layer not only have the same function as epithelium, the formation of a barrier and the regulation of transport between a cavity and the adjacent tissue, but also express E-cadherin and are located on a (fragmented) basement membrane. Although some studies could not report expression of E-cadherin in synovial tissue [29], our study is in line with a study by Trollmo and colleagues [30], describing E-cadherin expression in the lining layer of healthy synovial tissue, indicating the presence of molecules that mediate cell-to-cell adherence. As in epithelium, collagen IV, an important constituent of a basement membrane, was readily detectable in the lining layer of healthy synovium, seemingly forming a layer underneath the synovial lining cells [31]. Although some mesenchymal markers, such as vimentin and cadherin-11, are also present in the lining and sublining of healthy synovial tissue [29,32], expression of α-sma is absent, while expression of the epithelial marker E-cadherin is present. Together with its function – lining of the joint cavity, ultrafiltration of synovial fluid and excretion of hyaluronan into the synovial fluid – these characteristics indicate that synovial tissue represents an epithelial-like tissue. In synovial tissue from RA patients, E-cadherin staining occurs in both the lining and the sublining adjacent to the lining. This same staining pattern is found for collagen type IV. This observation implies that, in RA patients, cells from the lining layer start proliferating, but maintain part of an epithelial phenotype when they migrate more into the sublining. Expression of collagen type IV has also been found by others [31,33]. Rinaldi and colleagues [33], however, found a decrease in collagen IV expression, which correlated with the grade of inflammation of the rheumatoid synovia. This decrease in collagen type IV indicates the destruction of a basement membrane, a feature of EMT/fibrosis. In addition, they found that collagen type IV was expressed by FLSs. However, collagen type IV expression could not be downregulated by TGF-β *in vitro *[33].

The synovial membrane in RA patients shows features of both tumours and fibrotic tissue, as a tumour-like hyperplasia of the synovial lining and a fibrotic deposition of extracellular matrix are present. In diseases such as renal fibrosis and carcinomas, transition of an epithelial phenotype to a mesenchymal phenotype plays an important role in disease pathology, as it alters not only the phenotype, but also the migratory potential of the cells. Although we did not analyse the biological consequences of the alterations induced in FLSs after exposure to SF, it has been described that loss of E-cadherin and a change in integrin lead to the loss of cell-cell adhesion [17]. In addition, increases in α-sma and the cytoskeletal rearrangement provide tools for gaining motility. Therefore, we propose that the EMT phase or the number of synovial cells that have undergone EMT is indeed correlated with the infiltration of synovial cells into cartilage. The findings of this study, combined with a previous observation describing how invasiveness of FLSs is patient specific – as the difference in invasiveness between patients is greater than the difference between two joints from one patient [34] – suggest that a regulated EMT-like process might play a role in development of an arthritic synovium, rather than a random process of tumour-like alterations caused by the presence of p53 mutations in RA synovium [8,9,34].

A myofibroblast marker, α-sma, was found in the lining of half of the RA patients, indicating that, in these RA patients, synovial cells have a myofibroblastic phenotype, the same cells responsible for collagen accumulation in fibrosis. These data are supported by a finding by Aidinis and colleagues [35], who also found stress fibres in RA synovium, indicating that myofibroblasts might be present. Our study shows that SF from RA patients is able to induce α-sma expression in cultured healthy FLSs. These data indicate that factors present in the synovial fluid from RA patients are able to induce a transformation to myofibroblasts. Although both SF and TGF-β had an effect on the healthy FLSs, the mechanism seems to be different. After TGF-β stimulation, primarily collagen type I and TLH expression are upregulated; after SF stimulation, α-sma and TLH expression are upregulated. In the healthy FLSs, collagen I expression rather than α-sma expression was induced after stimulation with TGF-β. These findings seem to be in discordance with a study in which it was shown that cultured FLSs from RA patients transformed from α-sma negative cells to α-sma positive cells after stimulation with TGF-β [36]. In that study, however, only high levels of TGF-β were able to induce α-sma expression. In our study, we used a level of TGF-β previously found present in SF from RA patients [28]. Therefore, the use of synoviocytes from healthy individuals combined with lower concentrations of TGF-β to stimulate these synoviocytes might explain the absence of α-sma induction by TGF-β in our synoviocytes. Although in this study TGF-β was not able to induce α-sma mRNA expression, SF was able to induce α-sma expression in the healthy synoviocytes in the relatively short time frame analysed. This indicates that, besides TGF-β, other factors can play an additional role in the induction of alteration of phenotype in RA. Although the effects of many cytokines have not been studied yet, it has been described that the formation of myofibroblasts results from a combination of cellular fibronectin extra domain A (ED-A) with TGF-β [37]. Fibronectin ED-A is an isoform of fibronectin and has elevated levels in RA synovial tissue [38]. Therefore, it is possible that TGF-β plays a more important role in patients, because the cellular fibronectin ED-A present in the synovial tissue will enhance TGF-β activity.

In addition, the combination of the accumulation of receptor for advanced glycation endproducts (RAGE) ligands in SF from RA patients [20,39-44] and the ability of AGEs to induce EMT in kidney fibrosis via RAGE triggering independently of TGF-β [22,23] indicates that triggering of the RAGE could play a role in the induction of EMT/fibrosis in RA patients.

Previously, BMP-7 has been shown to counteract the action of TGF-β in inducing EMT and to reverse chronic renal injury [24]. This study shows that BMP-7 is also able to suppress alteration in FLSs induced by SF from RA patients. Therefore, BMP-7 therapy is potentially not only interesting for RA patients for its beneficial effects on cartilage and bone metabolism [45], but also for its role in maintaining a quiescent phenotype of the synovial lining layer.

## Conclusion

Like epithelium, the synovial lining layer forms a barrier between a cavity and the adjacent tissue. However, while synovial fibroblasts do express E-cadherin, other features of epithelium, such as the formation of a continuous cell layer and the presence of tight junctions and desmosomes, are not found. Therefore, the synovial lining can be called epithelial-like rather than a real epithelium. Nevertheless, this does not mean a process resembling EMT/fibrosis cannot occur; EMT has been shown in the endocardium, a tissue also lacking desmosomes [46]. In addition, our data show the presence of E-cadherin and collagen type IV and supports the finding that synovial tissue can undergo a process resembling EMT/fibrosis. In this process the resting synovial lining layer becomes hyperplastic and acquires an altered phenotype, including an increased invasive mobility, thereby contributing to joint destruction in patients with RA. To inhibit or reverse this process, administration of BMP-7 could be an interesting option for treatment of RA.

## Abbreviations

β2M = β2-microglobulin; AGE = advanced glycation endproducts; BCIP = 5-bromo-4-chloro-3-indolyl-phosphatase; BMP = bone morphogenetic protein; ED-A = extra domain A; EMT = epithelial to mesenchymal transition; FLS = fibroblast-like synoviocyte; HRP = horse radish peroxidase; NBT = 4-nitro blue tetrazolium chloride; PBS = phosphate-buffered saline; RA = rheumatoid arthritis; RAGE = receptor for advanced glycation endproducts; SF = synovial fluid; sma = smooth muscle actin; TGF = transforming growth factor; TLH = telopeptide lysylhydroxylase.

## Competing interests

The authors declare that they have no competing interests.

## Authors' contributions

MMCS acquired murine material, carried out immunohistochemistry, cultures and real-time PCR and was responsible for statistical analysis, participated in the study design and drafted the manuscript. TCAT was involved in acquisition of patient material and immunohistochemistry. GvdP was involved in conception of the study and participated in the design of the study. CL was involved in conception of the study and participated in the design of the study. CPJV was involved in acquisition of patient material. JDG gave general support and participated in the design of the study. ACG-DG was involved in the design of the study and interpretation of data. M CDR was involved in the design of the study and interpretation of data. BJW was involved in the design of the study and interpretation of data and gave technical support. TWJH was involved in conception of the study, participated in the design of the study and was involved in interpretation of the data. REMT was involved in conception of the study and participated in the design of the study and helped to draft the manuscript.

## Figures and Tables

**Table 1:** Primers for real-time PCR

Name	Forward primer	Reverse primer
CollIA2	5'-CAAGGACAAGAAACACGTCTGGCTAGGAGAAA-3'	5'-CAGGCGCATGAAGGCAAGTTGGGTAG-3'
α-sma	5'-CGTGTTGCCCCTGAAGAGCAT-3'	5'-ACCGCCTGGATAGCCACATACA-3'
TLH	5'-TTAAAGGAAAGACACTCCGATCAGAGATGA-3'	5'-AATGTTTCCGGAGTAGGGGAGTCTTTTT-3'
β_2_M	5'-TCTTGTACTACACTGAATTCACCCCCACTGA-3'	5'-ATCCAAATGCGGCATCTTCAAACCTC-3'
Col IV	5'-GCTCACCAGACCAGTGGGT-3'	5'-TCACCTTTAGGTGCTG-3'

β2M, β2-microglobulin; CollIA2, collagen type I alpha 2 chain; sma, smooth muscle actin; TLH, telopeptide lysylhydroxylase.

**Table 2:** Molecular beacons for real-time PCR

Name	Sequence
CollIA2	5'-FAM-cgtgccGGCAGCCAGTTTGAATATAATGTTGAAGGAggcacg-DABCYL-3'
α-SMA	5'-FAM-cgtcgCCAAGGCCAACCGGGAgAAAATGACgcgacg-DABCYL-3'
TLH	5'-cgtgcgCGTGATAAACTGGATCCTGATATGGCTCTTcgcacg-DABCYL-3'
β_2_M	5'-FAM-cgtgcCCTGCCGTGTGAACCATGTGACTTTGgcacg-DABCYL-3'

β2M, β2-microglobulin; CollIA2, collagen type I alpha 2 chain; DABCYL, (4-dimethylaminophenyl)benzoic acid; FAM, 6-carboxyfluorescein; sma, smooth muscle actin; TLH, telopeptide lysylhydroxylase.
